# Histone Acylation beyond Acetylation: Terra Incognita in Chromatin Biology 

**DOI:** 10.22074/cellj.2015.506

**Published:** 2015-04-08

**Authors:** Sophie Rousseaux, Saadi Khochbin

**Keywords:** Spermatogenesis, X Inactivation, HDAC, HAT, Bromodomain

## Abstract

Histone acetylation, one of the first and best studied histone post-translational modifications (PTMs), as well as the factors involved in its deposition (writers), binding (readers) and removal (erasers), have been shown to act at the heart of regulatory circuits controlling essential cellular functions. The identification of a variety of competing histone lysine-modifying acyl groups including propionyl, butyryl, 2-hydroxyisobutyryl, crotonyl, malonyl, succinyl and glutaryl, raises numerous questions on their functional significance, the molecular systems that manage their establishment, removal and interplay with the well-known acetylation-based mechanisms. Detailed and large-scale investigations of two of these new histone PTMs, crotonylation and 2-hydroxyisobutyrylation, along with histone acetylation, in the context of male genome programming, where stage-specific gene expression programs are switched on and off in turn, have shed light on their functional contribution to the epigenome for the first time. These initial investigations fired many additional questions, which remain to be explored. This review surveys the major results taken from these two new histone acylations and discusses the new biology that is emerging based on the diversity of histone lysine acylations.

## Introduction

Histone lysine acetylation was the first histone acylation identified in the sixties and hence it is the first histone post-translational modification ( PTMs ) for which molecular systems, mediating its establishment, removal and, later on, its recognition, were discovered and thoroughly investigated (1). Histone-deacetylase ( HDAC ) inhibitors were also among the first identified small molecules "epigenetic" drugs used to explore the cellular regulatory circuits involving histone acetylation. These inhibitors are now showing their efficacy in a series of unrelated pathologies including cancer (2). 

Several years ago, a pioneering work reported the important discovery that histones could bear lysine propionylation (K_Pr_) and butyrylation (K_Bu_) and also that some of the well-known acetyltransferases could be involved in these modifications (3). 

These findings represented a turning point in our understanding of how histone marks, and more particularly histone acetylation, could signal chromatin activities. Indeed, most of the identified histone K_Pr_and K_Bu_sites are also known to be acetylated (3,4). Therefore these studies raised questions on the functional similarities and differences between acetylation and these two newly found acylations. However, up to now, there has been no clear answer to these questions. 

The situation became even more complicated after a second wave of histone PTM discovery published in several recent articles reporting the 2 identification of additional acylated lysine sites including crotonylation (K_Cr_), 2-hydroxyisobutyrylation (K_Hib_), succinylation (K_Su_), malonylation (K_Ma_) and glutarylation (K_Glu_) (4,7). 

**Fig.1:**
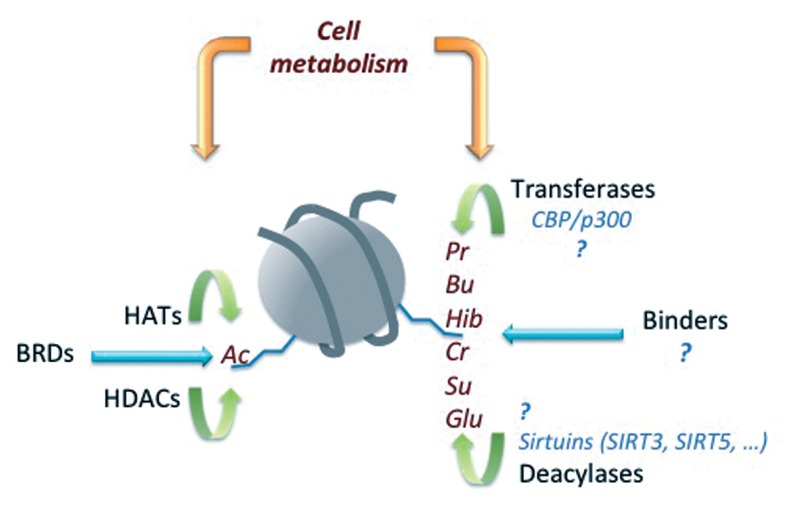
Molecular systems orbiting around histone acetylation have been the centre of comprehensive investigations leading to the identification and functional characterization of the three major classes of actors involved in generating [acetyltransferases ( HATs )], reading [bromodomains ( BRDs )] and erasing [deacetylases ( HDACs )] signalling to chromatin based on acetylation. In contrast, our knowledge of the molecular machinery managing signalling through histone acylations other than acetylation is very poor and only a few enzymes involved in their establishment and removal have been identified so far. All the acyl group donors are generated through cell metabolism and hence a critical question to address is how cell metabolism drives all these modifications and how it imposes a specific choice on the use of acyl group. Ac; Acetylation, Pr; Propionylation, Bu; Butyrylation, Hib; 2-hydroxyisobutyrylation, Cr; Crotonylation, Su; Succinylation, Glu; Glutarylation and CBP/p300; CREB-binding protein/EP300.

Since all the newly discovered acylations could potentially compete with histone lysine acetylation, questions have emerged such as how the choice of histone acylation is made, which enzymes are involved and what the functional interplay is between these modifications in general and more specifically with histone acetylation (Fig.1). 

However, two of these studies, including a detailed genome-wide mapping of histone K_Cr_and K_Hib_sites respectively in differentiating mouse spermatogenic cells brought the first hints concerning the functional significance of these new histone PTMs relative to the corresponding histone acetylation (4,5). 

## Spermatogenesis: an ideal system to assign a functional significance to unknown histone PTMs

Spermatogenesis is a specific differentiation program that aims at producing highly specialized haploid cells capable of transporting the male genome out of the father’s organism into the female genital track to meet the oocyte (8). 

The requirement for these cells to leave their organism of production is backed by very specific gene expression programs that are mostly silent in all somatic adult tissues but become active only in male germ cells in an orderly manner (9,11). Progenitor adult stem cells, or spermatogonia, continuously feed a flow of cells, which will undergo meiosis followed by a post-meiotic differentiation process. Distinct and specialized gene expression programs are turned on during each of the three major stages of spermatogenesis, which include self-renewing mitotically dividing stem cells ( spermatogonia ), meiotically dividing cells ( spermatocytes ) and haploid cells ( spermatids ). The latter cells undergo a complete metamorphosis to become "swimming" mature spermatozoa. Defined categories of genes become in turn active or silent during each of these periods of spermatogenesis (10). Additionally, there are specific chromosome-wide transcriptional regulatory events that make this system a particularly rich one in terms of the variety of gene expression regulatory events. Indeed, during meiosis, the sex chromosomes become transcriptionally inactive, following a phenomenon known as meiotic sex chromosome inactivation ( MSCI ) while, after meiosis, a fraction of the X/Y-linked genes escapes the inherited general silencing and is reactivated (12,13). 

Genome-wide mapping of particular histone marks in both meiotic and post-meiotic cells associated with whole genome gene expression profiling, allows assigning a functional significance to a given mark. Indeed, at first, like in many other cellular systems, histone mark distributions are considered as a function of genome annotations: intergenic regions, gene promoters, enhancers, transcriptional start sites ( TSS ) exons, introns and etc. Once a significant association of a given histone PTM with gene regulatory elements, such as enhancers, TSS and etc, is observed, in a second step, it becomes possible to consider the transcriptional status of the corresponding genes. Taking into account both meiotic and post-meiotic cells with different gene expression profiles, one can easily associate a particular histone mark with increased or decreased gene activities (14). When these studies consider a given histone acylation in parallel with histone acetylation, it becomes possible to monitor the interplay between histone acylation and acetylation (4,5,15). 

Additionally, a unique event during the postmeiotic phases of spermatogenesis is the almost genome-wide histone eviction followed by repackaging of the genome with small basic non-histone proteins. Interestingly, it has been observed that, in different species, histone removal is associated with a genome-wide histone hyperacetylation. This system constitutes another interesting readout for comparisons between histone acetylation and other histone acylations (16). 

## Histone crotonylation and 2-hydroxyisobutyrylation: two specific histone marks indicating active

genes The genome-wide mapping of histone lysine K_Cr_in differentiating spermatogenic cells shows an enrichment of this mark at gene TSSs. 

Interestingly, a specific category of male germ cell-specific genes is associated with an increase in histone crotonylation between meiotic and post-meiotic stages. Further investigations have revealed that they are genes, which become active after meiosis and are specifically expressed in haploid male germ cells. Among these late activated genes, of particular interest are the X-linked genes ( localised on the X chromosome ) for which this increase in histone crotonylation precisely marks genes that escape inactivation. Under the same conditions, no particular association was observed between variations in lysine acetylation (K_Ac_) and changes in gene expression (4). Later on, an independent study confirmed the above reported finding and further demonstrated that histone crotonylation specifically marks X-linked genes that escape inactivation (17). 

Similar genome-wide analyses were performed to explore histone K_Hib_, but this time on a specific lysine site of histone H4, K8, and revealed a situation somewhat similar to that described above for histone K_Cr_. H4K8_Hib_was found enriched at the TSS of active genes and in post-meiotic cells, the presence of H4K8_Hib_at gene TSS appeared to be a better indicator of transcriptional activity than H4K8_Ac_. Interestingly, considering X-linked genes, the majority of genes escaping inactivation in post-meiotic cells, which had been previously identified as labelled with histone K_Cr_, were also marked with H4K8_Hib_. However, the TSS of these genes was not associated with H4K8_Ac_(5). 

Therefore these analyses reveal that the two newly identified histone acylations, similar to histone acetylation, are associated with transcriptionally active genes. However, the specific situation of post-meiotic genes labelled with both histone K_Cr_and K_Hib_, in particular X-linked genes that escape the inherited MSCI, suggests a specific role for these acylations that may not be shared by acetylation. 

## Hot topics on the functional significance of histone acylations

Detailed study of the two newly discovered histone acylations, K_Cr_and K_Hib_, suggests that, although both could mark active genes in a similar way as K_Ac_, they might also act under specific circumstances, where histone acetylation would not function properly. In subport of this hypothesis one could consider the existence of enzymes involved in their establishment and removal that are different from those mediating histone acetylation. The above reported investigations have indeed shown that histone crotonylation behaves differently from acetylation with respect to class I and II HDACs, which are efficient deacetylases but poor decrotonylases (4). Additional investigations revealed that HDAC3 (18) and Sirt1 and Sirt2 (19) could decrotonylate histone peptides *in vitro* to some extent. A new study, including ** assays, showed that Sirt3 is the major *in vivo* decrotonylase, specifically involved in the regulation of H3K4 _Cr_(20). Sirt3 being essentially a mitochondrial enzyme, the remaining question is to what extend the residual amounts of nonmitochondrial enzyme could regulate chromatin crotonylation. 

Regarding K_Hib_, a previous work showed that, 4 when this mark is present on free histones, it could be erased *in vitro* by HDAC1, 2 and 3 (5). However, the efficiency of these reactions compared to deacetylation by the same enzymes and whether they could take place *in vivo* remain to be investigated. 

There is also information on another lysine acylation, K_Glu_, which shows resistance to all HDACs except Sirt5 (6). 

A particular characteristic of post-meiotic spermatogenic cells is that, prior to histone eviction, they undergo a genome-wide transcriptional repression (21,22). This could be associated with a gradual instauration of a general transcriptional repressive environment and a need for the genes that remain active in these cells to use "stronger" active histone marks, meaning histone PTMs that would be more stable and would better resist an enzymatic removal by at least some of the common deacetylases. 

Although at the genomic scale the presence of such an epigenetically-driven repressive environment has not been clearly established in early post-meiotic spermatogenic cells, at the sex chromosomes scale, there is no doubt about the existence of a repressive environment. First, most of the sex chromosome-linked genes inactivated during meiosis remain silent in postmeiotic cells and second, sex chromosomes locate adjacent to the repressive heterochromatic chromocenter (15). Therefore, it is possible to speculate that in such an environment acylations other than acetylation could be required to overcome and resist gene silencing. 

Indeed, in many cases, class I and II HDACs contribute to gene silencing, mostly by deacetylating chromatin. Interestingly, as mentioned above, histone K_Cr_and K_Glu_are not good substrates for class I and II HDACs and need the class III enzymes, more specifically Sirt3 and Sirt5, to be removed (6,20). As a consequence, since all HDACs contribute to the instauration of a repressive chromatin, histone crotonylation as well as glutarylation offer a better chance to resist repression than histone acetylation, a mark that could be removed by all these enzymes. The same reasoning could apply for histone K_Hib_. However, in this case, a strong conclusion on this mark’s stability awaits a better characterization of class I/II HDACs and Sirtuins involved in its removal. 

Another interesting question concerns enzymes involved in the establishment of histone acylation. With this regard, we have limited knowledge. 

The only available information is that the histone acetyltransferases ( HATs ) CREB-binding protein/ EP300 ( CBP/p300 ) can mediate histone propionylation and butyrylation (3). Also these enzymes probably catalyse the establishment of additional acyl groups such as succinyl and glutaryl (6). The questions are whether these enzymes catalyse other actylations and also whether other known HATs would be capable of mediating these modifications. 

As discussed above, the knowledge of the molecular systems controlling the establishment and removal of histone acylations beyond acetylation could represent an essential step for a better understanding of the functional significance of these new marks. 

The answer to this question leads to another question: if the same enzymes mediate multiple histone acylations, what drives the choice on the acyl group to be transferred? 

If a particular HAT catalyses several distinct acylations, then the availability of the donor acyl group should influence the outcome of the reaction. 

Indeed, it is believed that Co-enzyme A is the carrier and donor of most of the acyl groups (4,5,23,25). Since Acyl-CoAs are generated following different metabolic pathways, their availability should directly depend on the state of cell metabolism, which should in turn have influence on the nature of the acyl groups used to modify histones (Fig.1). 

## Conclusion

The recent breakthrough discovery of a large panel of new histone PTMs should definitely change our vision of the epigenome. Indeed, about twenty years of gradually intensifying research on acetylation and thousands of publications were dedicated to only one of the many possible histone lysine acylations. Now the question is whether all the discoveries about the molecular signalling relying on histone acetylation could also stand and apply if another acyl group replaces the acetyl. The next question is whether specific signalling to chromatin depends on each of these PTMs or if many or all the acylations could be "functionally" grouped in a category of “non-acetyl” group. The latter hypothesis would imply that all types of acylations other than acetylation, would have the same functional consequence, which would be different from acetylation. Therefore, a metabolic shift increasing the ratio of acyl-CoA/ acetyl-CoA would favour histone "non-acetyl" acylation with a unique functional consequence, independent from the used acyl group. 

In support of this hypothesis, the two studied acylation marks, crotonylation and 2-hydroxyisobutyrylation, were found functionally equivalent, i. e. positively associated with gene activity and more resistant than acetylation to gene repressive mechanisms and both could be predominantly used when cell metabolism generates more crotonyland 2-hydroxyisobutyrylCoA than acetyl-CoA. 

The points discussed above are just only several possibilities among many others and a better vision of the contribution of histone acylations to the epigenome would need much more investigations on the cellular and molecular mechanisms involving these new histone PTMs.
